# Dynamic Thiol Disulphide Homeostasis in the Follow-Up of the Prognosis of Patients Treated for COVID-19 in the Intensive Care Unit

**DOI:** 10.7759/cureus.27542

**Published:** 2022-07-31

**Authors:** Erdoğan Duran, Abdullah Taşkın, Basak Pehlivan, Hakim Çelik, Veli Fahri Pehlivan, Seyhan Taşkın

**Affiliations:** 1 Department of Anesthesia and Reanimation, Faculty of Medicine, University of Harran, Sanliurfa, TUR; 2 Department of Nutrition and Dietetics, Faculty of Medicine, University of Harran, Sanliurfa, TUR; 3 Department of Physiology, Faculty of Medicine, University of Harran, Sanliurfa, TUR

**Keywords:** thiols, prognosis, intensive care units, covid-19, coma

## Abstract

Introduction: Evaluation of the prognosis in the early period of intensive care patients and arranging the treatment accordingly is of vital importance. In the present study, we investigated whether serum thiol/disulphide concentration can be used in the follow-up of prognosis in the early period in patients with COVID-19 under intensive care.

Methods: The study included 25 patients [their ages were between 19 and 92; 10 (40%) were male and 15 (60%) were female] who were diagnosed with COVID-19 and treated in the intensive care unit (ICU). The patients were followed for four weeks. On the first, third, and fifth days of intensive care treatment, venous blood samples were taken from the patients to analyze the thiol/disulphide parameters, and coma scores were calculated. Statistical analyses were conducted to evaluate the relationship between thiol/disulphide levels and the prognosis of COVID-19 patients.

Results: At the end of the four-week follow-up of the patients included in the study, 9 were discharged and 16 died. In patients who died, the relationship between thiol/disulphide homeostasis parameters and coma scores was not statistically significant. Meanwhile, in discharged patients, the relationship between disulphide concentration, total thiol, and coma scores was statistically significant.

Conclusion: The relationship between thiol/disulphide homeostasis and coma scores in COVID-19 patients treated in the intensive care unit may help to evaluate the prognosis of the disease in the early period, thus the effectiveness of medical intervention.

## Introduction

COVID-19 caused by SARS-CoV-2 is characterized by clinical conditions that increase mortality risk [1]. Complications such as pneumonia, cardiovascular dysfunction, acute respiratory distress syndrome (ARDS), and multiple organ failure are the most serious problems caused by COVID-19. These patients frequently require intensive care unit (ICU) treatment and are at high risk of death [2]. These complications are attributed to systemic inflammatory responses, cytokine storms, and autoimmune activation [3].

Protein thiols and low-molecular-weight plasma thiols are involved in many cellular processes, such as the regulation of protein function, antioxidant defense, apoptosis, signalling mechanisms, and immune response [4-6]. Thiols are highly sensitive to oxidation due to the -SH groups in their structure. Oxidized thiols are the most important class of dynamic redox-sensitive covalent bonds formed between two thiol groups. Since these molecules constantly interact with almost all physiological oxidants, they are considered the fundamental antioxidant buffers [6-8]. Thiol/disulphide homeostasis, an important indicator of the cellular redox state, is impaired in the pathogenesis of various disorders [6,9].

During the clinical follow-up of patients with COVID-19 treated in ICUs, routine hemogram-biochemistry (C-reactive protein [CRP], ferritin, lactate dehydrogenase [LDH], and D-dimer, among others), radiography, and other examinations are performed to obtain information on the clinical course [10]. The disease course and progression toward death must be determined at an earlier stage to select the appropriate treatment. In the present study, we aimed to evaluate the serum thiol/disulphide homeostasis in follow-up prognosis in the early period among patients with COVID-19 under intensive care.

## Materials and methods

Study design

The present prospective study was approved by the Ministry of Health and the Harran University Faculty of Medicine Clinical Research Ethics Committee (Approval Dates: October 22, 2021 and November 01, 2021, Session No. 19). Voluntary informed consent was obtained from all patients included in the study or their first-degree relatives. In accordance with the Declaration of Helsinki, all patient data was anonymized.

Twenty-five patients who were diagnosed with COVID-19 and treated in the COVID-19 ICU of Harran University Hospital in November and December 2021 were included. The patients were followed for one to four weeks. The real-time polymerase chain reaction (RT-PCR) test results of patients were positive, and their radiographs showed a diffuse infiltrative ground glass appearance in both lungs, consistent with COVID-19.

Clinical and biochemical data

Patients' age, sex, pregnancy status, comorbidities, ventilator support, coma scores (Acute Physiologic and Chronic Health Evaluation-APACHE II score, Glasgow Coma Scale/Score-GCS, Sequential Organ Failure Assessment-SOFA), sedation status, COVID vaccine status, treatments, mortality rates, and discharge status were all recorded. Patients who received treatment for less than five days, patients with a ratio of arterial partial pressure of oxygen to fraction of inspired oxygen (PaO_2_/FiO_2_) >300 mmHg, autoimmune disease, PCR negative afterward, tocilizumab treatment, and patients <18 years of age were excluded from the study. There were no patients who were given Remdesivir.

On the first, third, and fifth days of treatment, venous blood samples were taken at the same time every day to determine biochemical parameters, complete blood count, and thiol-disulphide homeostasis levels. For biochemical and thiol/disulphide homeostasis measurements, the blood samples were centrifuged at 1500 ×g for 10 min. Serum samples were stored at −86 °C until they were analyzed. Complete blood count with Abbott Alinitiy hq analyzer (USA); serum albumin, urea, creatinine, CRP, ferritin, and LDH with Atellica solution analyzer (Siemens Healthineers, Germany); D-dimer levels with the Sysmex CS-2100i instrument (Sysmex, Japan) were analyzed.

Testing process

The thiol-disulphide homeostasis was analyzed using the spectrophotometric method of Erel and Neselioglu, which is based on the reduction of dynamic disulphide bonds (-S-S-) to reactive thiol groups in the presence of sodium borohydride (NaBH_4_) [11]. The concentrations of total thiol and native thiol (-SH) in serum samples were determined using Ellman and modified Ellman reagents [12]. The number of -S-S- was calculated by subtracting the -SH content from the total thiol content and dividing the difference in half. Thiol oxidation reduction [(-S-S-) × 100 / (-SH)], Oxidized thiol [(-S-S-) × 100 / total thiol)], and reduced thiol [(-SH × 100) / total thiol] ratios were also calculated.

Statistical analysis

Statistical analyses were carried out using the Statistical Package for the Social Sciences 25.0 package programme (IBM SPSS Inc., Chicago, IL, USA). The Shapiro-Wilk test was used to determine the normality of the data distribution. Normally distributed numerical variables were expressed as mean ± standard deviation and non-normally distributed data as median (interquartile range). Numbers (n) and percentages (%) were used to represent categorical variables (percent). In parametric assumptions, the repeated measures analysis of variance (ANOVA) test was used for statistical analysis of dependent variables measured at three different times. The paired-samples T-test was used to investigate the relationship between two dependent variables. For non-parametric assumptions, the Friedman ANOVA test was used to perform a statistical analysis of dependent variables measured at three different times. The Wilcoxon test was used to investigate the relationship between two non-parametric dependent variables. The relationship between thiol-disulphide parameters and coma scores was examined using Spearman correlation analysis. In the analysis, the confidence interval (CI) was set at 95%. p <0.05 was regarded as statistically significant. For 25 patients, post-HOC power analysis was calculated as 0.93 for 0.05 error margin and 0.59 effect size, based on the disulphide value. Power analysis was done with the G*Power3.1 program.

## Results

The patients were between the ages of 19 and 92 (62.6±18.4) years; 10 (40%) were male and 15 (60%) were female. Of the patients, 12 (48%) were unvaccinated and 13 (62%) were vaccinated. Of those vaccinated, two were previously vaccinated with a single dose, eight with two doses, one with three doses of CoronaVac-Sinovac, and two with a single dose of Pfizer-BioNTech BNT162b2. 56% (n = 14) of the patients were intubated. Six of the intubated patients (42.8%) were previously vaccinated with at least two doses of CoronaVac-Sinovac. Twenty (80%) of the patients had comorbidities. Of these, 16 (80%) had at least one disease (such as diabetes, asthma, chronic obstructive pulmonary disease, cerebrovascular disease, coronary artery disease, and heart rhythm disorders). In addition to hypertension, two patients (8%) were pregnant, and three patients (12%) had no additional disease. The PCR positivity of the patients ranged from 1 to 28 days. Seven (28%) of the patients were discharged; 1 (4%) was transferred from the COVID ICU to the service; 1 (4%) was taken to the general ICU after PCR negativity; and 16 (64%) died.

At three different times, the laboratory findings, coma scores, and thiol/disulphide levels of patients who died from COVID-19 are shown in Table 1. White blood cell (WBC), urea, creatinine, LDH, ferritin, and D-dimer levels increased in those who died from COVID-19, while albumin and CRP levels decreased. Unlike other biochemical parameters, the decrease in CRP over time was statistically significant (p = 0.006). While there was no significant change in SOFA levels over time, APACHE II scores increased while GCS decreased. GCS decreased in a statistically significant way (p = 0.016). Changes in laboratory findings and coma scores were linked to the prognosis of COVID-19 patients who died. The changes in serum thiol/disulphide levels on the first, third, and fifth days of hospitalization in the intensive care unit of the deceased patients were not statistically significant.

**Table 1:** Clinical characteristics and thiol/disulphide parameters of individuals who died from COVID-19 in ICU Data are expressed as mean±standard deviation and median (interquartile range) where appropriate. ^α^There is a difference between day 1 and day 3 (p ˂0.05); *There is a difference between day 1 and day 5 (p ˂0.05). APACHE II: Acute Physiology and Chronic Health Evaluation II; SOFA: Sequential Organ Failure Assessment; GCS: Glasgow Coma Scale; NT: native thiol; TT: total thiol; DB: disulphide bond; RT: reduced thiol; OT: oxidized thiol; TOR: thiol oxidation-reduction; COVID-19: Coronavirus disease 2019; ICU: intensive care unit; WBC: white blood cell; LDH: lactate dehydrogenase; CRP: C-reactive protein.

Died from COVID-19 ICU (n=16)				
	Day 1	Day 3	Day 5	p-value
Haemoglobin, mg/dL	12.7 (4.7)	11.2 (4.3)	11.1 (4.3)	0.383
WBC × 10^3^/μL	11.3 (7.8)	11.5 (4.8)	13.1 (10.12)	0.926
Albumin, g/dL	3.20 (0.9)	3.10 (0.5)	2.9 (0.7)	0.423
Urea, mg/dL	94.1 (107.0)	117.7 (96.3)	124.1 (146.5)	0.102
Creatinine, mg/dL	1.10 (1.6)	1.40 (1.8)	1.40 (3.2)	0.145
LDH, U/L	383.3 (253.0)	345.0 (397.0)	431.0 (345.0)	0.116
Ferritin, µg/L	118.2 (632.5)	296.9 (839.0)	362.3 (808.7)	0.794
CRP, mg/L	8.48 (11.6)	4.10 (6.7)	2.39 (10.3)	0.006 ^α^
D-dimer, mg/dL	1.96 (3.92)	1.46 (2.7)	2.36 (5.3)	0.199
APACHE II	15.0 (17.7)	15.0 (21.0)	23.0 (24.5)	0.449
SOFA	9.5 (14.2)	11.5 (21.2)	11.5 (21.2)	0.135
GCS	13.0 (12.0)	3.0 (4.5)	3.0 (6.0)	0.016^ α, *^
NT (µmol/L)	260.85 ± 70.55	283.32 ± 65.03	264.51 ± 68.9	0.593
TT (µmol/L)	502.73 ± 100.67	506.76 ± 90.13	639 ± 276.25	0.112
DB (µmol/L)	111.41 (69.51)	106.46 (56.81)	173.5 (188.39)	0.144
RT (%)	52.48 ± 12.65	57.57 ± 15.11	47.37 ± 18.33	0.231
OT (%)	23.75 ± 6.32	21.21 ± 7.55	26.31 ± 9.16	0.231
TOR (%)	49.48 (35.55)	39.18 (24.86)	68.13 (55.89)	0.269

Likewise, Table 2 shows the laboratory findings, coma scores, and thiol/disulphide levels of patients discharged from COVID-19 at three different times. The CRP, WBC, and LDH levels of the discharged COVID-19 patients decreased over time. The WBC level was statistically significant between the groups (p=0.018). There were no significant changes in other biochemical parameters. There was no significant change in the coma scores of the discharged patients. Disulphide, reduced thiol, oxidized thiol, and thiol oxidation-reduction levels in blood samples taken at different times of discharged patients were statistically significant (p=0.010, p=0.003, p=0.003, p=0.013, respectively). There is a difference between the first versus fifth days and the third versus fifth days of the mentioned parameters.

**Table 2:** Clinical characteristics and thiol/disulphide parameters of individuals who healthy discharge from COVID-19 in ICU Data are expressed as mean±standard deviation and median (interquartile range) where appropriate. *There is a difference between day 1 and day 5 (p ˂ 0.05); ^β^There is a difference between day 3 and day 5 (p˂0.05). APACHE II: Acute Physiology and Chronic Health Evaluation II; SOFA: Sequential Organ Failure Assessment; GCS: Glasgow Coma Scale; NT: native thiol; TT: total thiol; DB: disulphide bond; RT: reduced thiol; OT: oxidized thiol; TOR: thiol oxidation reduction; COVID-19: Coronavirus disease 2019; ICU: intensive care unit.

Healthy discharge from COVID-19 ICU (n=9)				
	Day 1	Day 3	Day 5	p-value
Haemoglobin, mg/dL	12.5 (2.8)	12.1 (3.9)	12.4 (2.9)	0.495
WBC ×10^3^/μL	12.0 (4.4)	13.3 (3.8)	9.3 (4.0)	0.018^*^
Albumin, g/dL	3.20 (0.7)	3.3 (1.0)	3.20 (0.9)	0.962
Urea, mg/dL	32.1 (66.3)	42.8 (66.3)	40.6 (77.0)	0.250
Creatinine, mg/dL	0.70 (0.2)	0.70 (0.2)	0.70 (0.3)	0.291
LDH, U/L	393.0 (332.0)	331.5 (247.0)	280.0 (180.0)	0.050
Ferritin, µg/L	558.8 (929.7)	811.0 (996.7)	443.9 (783.2)	0.867
CRP, mg/L	8.96 (11.5)	7.0 (8.7)	2.57 (5.8)	0.066
D-dimer, mg/dL	1.86 (2.0)	0.96 (1.9)	1.48 (1.2)	0.236
APACHE II	13.0 (9.0)	13.0 (9.0)	13.0 (9.0)	0.921
SOFA	13.0 (8.7)	13.0 (8.7)	12.0 (8.7)	0.368
GCS	15.0 (3.0)	15.0 (3.0)	15.0 (0.0)	0.368
NT (µmol/L)	223.55 (30.25)	296 (123.61)	253 (101.25)	0.139
TT (µmol/L)	435.8 ± 89.12	529 ± 142.88	597 ± 151.42	0.081
DB (µmol/L)	98.97 ± 47.26	111.29 ± 48.76	181.99 ± 55.06	0.010^*,β ^
RT (%)	56.01 ± 14.59	59.13 ± 8.01	39.32 ± 8.64	0.003^*,β^
OT (%)	21.99 ± 7.29	20.43 ± 4	30.33 ± 4.32	0.003^*,β^
TOR (%)	45.08 (39.38)	33.71 (20.54)	68.57 (33.88)	0.013^*,β^

There was no statistically significant relationship between thiol-disulphide homeostasis parameters and coma scores in deceased patients (Table 3).

**Table 3:** Comparison of correlation analysis for total thiol, disulphide levels, GCS, APACHE II in individuals who died from COVID-19 in ICU Spearman correlation analysis. APACHE II: Acute Physiology and Chronic Health Evaluation II; SOFA: Sequential Organ Failure Assessment; GCS: Glasgow Coma Scale; TT: total thiol; DB: disulphide bond; COVID-19: Coronavirus disease 2019; ICU: intensive care unit.

Died from COVID-19 ICU (n=16)						
			TT	GCS	APACHE II	SOFA
	DB	r	0.828	−0.054	0.080	−0.284
		p	˂0.001	0.739	0.641	0.239
	TT	r		−0.091	−0.015	0.052
		p		0.569	0.933	0.831
	GCS	r			−0.420	−0.414
		p			0.011	0.078
	APACHE II	r				0.289
		p				0.244

The correlation between disulphide levels in discharged patients and total thiol (r=0.859, p<0.001), GCS (r=0.442, p=0.027), and APACHE II (r=−0.453, p=0.026) scores, on the other hand, was statistically significant. The correlation between total thiol levels and GCS (r=0.411, p=0.042) and APACHE II (r=−0.430, p=0.036) scores was found to be statistically significant in the same group (Table 4).

**Table 4:** Comparison of correlation analysis for total thiol, disulphide levels, GCS, APACHE II in individuals who healthy discharge from COVID-19 in ICU Spearman corelation analysis. APACHE II: Acute Physiology and Chronic Health Evaluation II; SOFA: Sequential Organ Failure Assessment; GCS: Glasgow Coma Scale; TT: total thiol; DB: disulphide bond; COVID-19: Coronavirus disease 2019; ICU: intensive care unit.

Healthy discharge from COVID-19 ICU (n=9)						
			TT	GCS	APACHE II	SOFA
	DB	r	0.859	0.442	−0.453	−0.261
		p	˂0.001	0.027	0.026	0.253
	TT	r		0.411	−0.430	−0.210
		p		0.042	0.036	0.360
	GCS	r			−0.797	−0.672
		p			˂0.001	0.001
	APACHE II	r				0.718
		p				˂0.001

## Discussion

There is a delicate balance between oxidant and antioxidant mechanisms in the functional immune system, and one of the most sensitive indicators of this balance is thiol/disulphide homeostasis [13]. This study is one of the rare studies investigating its utility in terms of dynamic thiol/disulphide balance and clinical course in patients treated in the COVID ICU. We found that the dynamic thiol/disulphide balance was disrupted in the early period of the first five days of treatment of patients treated in the COVID intensive care unit, and this delicate balance could not be maintained. In this balance, the statistically significant change in disulphide direction in discharged patients, unlike patients who died, suggests that this may be an indicator of immune response. The correlation of disulphide increase, one of the thiol/disulphide parameters, with total thiol, coma scores (GCS and APACHE II) and clinical data in discharged patients supports our hypothesis. Therefore, we think that disulphide increases and total thiol levels, together with GCS and APACHE II scoring, can be used in the follow-up of the prognosis of COVID-19 disease.

In the study of Oliveira and Laurindo, it was stated that thiols are an independent risk factor for the severity of COVID-19, and glutathione levels were significantly reduced in these patients [9]. In addition, the decrease in thiol/disulphide ratio in extracellular fluids may play an important role in promoting the physical interaction of the virus with host cells in the airways [1]. In fact, one study found that increasing thiol reserves could reduce the risk of COVID-19 complications, and glutathione levels were linked to longer recovery times and worsening symptoms [14]. In conditions that produce high levels of oxidative stress, disulphide levels rise during the neutralization of reactive oxygen species by thiols. [13]. In our current study, the higher disulphide levels in discharged patients are thought to be due to a possible immune response. For this reason, this response in disulphide levels may indicate that the prognosis of the disease will improve. Although ICU admission criteria in the COVID-19 pandemic may differ depending on the severity of the epidemic wave and available resources, the main criteria are generally similar. Certain laboratory values such as CRP, D-dimer, LDH, urea, creatine, albumin, and WBC are examples of these, as well as criteria such as low oxygen saturation, hemodynamic instability, presence of ARDS, coma scores, and the need for mechanical ventilation [15,16]. The findings of the patients included in our study were consistent with these criteria. Especially, APACHE II, SOFA, and GCS scores were found to be associated with clinical course in all patients.

According to present knowledge, COVID-19 infection is more lethal in those with co-morbidities, and targeted therapy should be initiated as soon as possible. As a result, it is important to understand the course of the disease at an earlier stage, especially in these critically ill patients [15]. Inflammation and oxidative stress have a strong relationship [17]. According to one study, oxidative stress caused by an imbalance in the production of reactive oxygen radicals and antioxidants may be linked to the pathogenesis and consequences of COVID-19 [18]. Furthermore, it has been demonstrated that thiol/disulphide homeostasis, which represents the systemic redox state, can change in response to disease severity [11]. Total thiol and native thiol levels, in particular, have been identified as important predictors of disease severity in paediatric and adult COVID-19 patients [13]. Another study found that the severity of infection and thiol levels were inversely related [19]. Thiol concentrations have been reported to be a good biomarker in determining admission to the intensive care unit in relation to the severity of the disease [20]. However, there is a study that reports the inverse of these findings [16]. Although native thiol levels varied depending on the treatments given to those who died from COVID-19 and those who were discharged from COVID-19 in our study, total thiol levels increased in lockstep with the length of stay in the intensive care unit. The rise in total thiol levels in patients may represent the immune system's response. On the fifth day of admission to the COVID ICU, the gradual increase in disulphide level, which is another marker of thiol/disulphide homeostasis, is an indication of the gradual increase in oxidative stress. This increase was more pronounced in people who had recently been discharged. To maintain dynamic thiol-disulphide homeostasis, oxidants can form reversible dynamic disulphide bridges between thiol groups [11]. The presence and maintenance of this reversible, delicate balance may be useful in monitoring COVID-19's inflammatory stress responses. The rise in disulphide levels in the discharged group suggests that the free radicals produced by the immune response can be compensated for. The discharged group's total thiol pool effectively reversibly formed dynamic disulphide bonds. The fact that the oxide thiol levels in the discharged group were at their highest on the fifth day of hospitalization in the ICU indicates that homeostatic balance is effectively achieved.

The potential effects of thiol/disulphide homeostasis on clinical progression and prognosis in the COVID-19 disease process have been linked to high thiol concentrations or antioxidant capacity [13,20,21]. Unlike these studies, our findings suggest that elevated disulphide levels are associated with the COVID-19 prognosis. The correlation between disulphide levels and APACHE II, GCS, and SOFA scores, which help predict the clinical course of patients in the COVID ICU, validates our findings. Disulphide levels in patients discharged from the COVID ICU were found to be positively correlated with total thiol and GCS, and negatively correlated with APACHE II score. The increase in total thiol concentration and high disulphide levels indicate that the free radical increase caused by the viral agent is oxidized by thiols. It can be stated that high disulphide levels in conjunction with the given treatments indicate a favourable prognosis. Increasing dynamic disulphide levels with high total thiol levels can be evaluated together with coma scores and can provide information about the clinical course of patients hospitalized in the COVID-19 ICU.

Our study has some limitations. Our study was conducted in a single centre in a small group of patients treated in the COVID-19 ICU. Therefore, our results require validation in a larger cohort.

## Conclusions

The presence of a high total thiol and disulphide relationship is an indication that redox homeostasis is dynamically maintained. Dynamic maintenance of thiol/disulphide homeostasis is indicative of improvement in ICU patients. Since total thiol and high disulphide levels are associated with coma scores, they may be candidates for use in monitoring the prognosis of COVID-19 patients hospitalized in the ICU.
